# Development of the Immune System

**DOI:** 10.1155/S106474499700015X

**Published:** 1997

**Authors:** Anne Durandy

**Affiliations:** Institut National de la Santé et de la Recherche Médicale U429 Hôpital Necker-Enfants Malades 149 rue de Sèvres Cédex15 Paris 75743 France


Infectious Diseases in Obstetrics and Gynecology 5:93–97 (1997)
© 1997 Wiley-Liss, Inc.

### Development of the Immune System

**Anne Durandy**

*Institut National de la Santé et de la Recherche Médicale U429, Hôpital Necker-Enfants Malades, Paris, France*

The development of immune defense mechanisms begins early during fetal life but is not completely achieved at birth, leading to a peculiar susceptibility of the neonate to infectious diseases.[1,2] The earliest hematopoietic stem cells are detectable in the yolk sac around 6 weeks of gestation, but at that time they are only able to differentiate into myelomonocytic cells. Thereafter, the hematopoiesis takes place in the liver from the 7th week of gestation until the 6th month, thereafter in the bone-marrow. A unique precursor is at the origin of all hematopoitic cell lines.

### NORMAL SPECIFIC IMMUNITY DEVELOPMENT

A schematic representation of the T and B lymphocyte differentiation is shown in Figure 1.

### Cellular Immunity

Lymphoid cells with the surface phenotype of pro-T (CD7+) are found in the fetal liver by 7 weeks of gestation, and the fetal thymus is colonized by 8.5 weeks of gestation. Pro-T cells (from fetal liver in early gestation, thereafter from bone-marrow) are attracted into the thymus by chemotactic agents secreted by thymic epithelial cells.[3] Shortly after, thymocytes express the marker CD2, as well as both markers CD4 and CD8 (pre-T cells). Then, the pre-T cells acquire the specific T-cell antigen receptor (TCR) associated with the complex CD3 and express either CD4 or CD8 molecules (mature T cells). The TCR which results from stochastic gene rearrangements specifically recognizes antigenic peptides which have been processed by antigen presenting cells only if presented by self HLA molecules; the diversity of the TCR repertoire is progressively acquired by positive (leading to amplification of T-cell clones able to recognize foreign antigens) and negative (allowing the destruction of autoreactive T-cell clones) selection mechanisms occuring in the thymus by close contact of pre-T lymphocytes with dendritic and epithelial cells.[4,5] Mature CD4+ T cells recognize by their TCR foreign antigenic peptides presented by HLA class II molecules, expressed on B cells and monocytes, and, by the way, are essentially helper T cells; in contrast, mature CD8+ T cells recognize foreign antigenic peptides presented by HLA class I molecules and, thus, are essentially cytotoxic/suppressor T cells. CD4+ T cells represent 65% and CD8+ 35% of mature (CD3+) T cells. Mature T cells are able to go out of the thymus, to circulate in the blood and to reach the lymphoid organs (spleen and lymph nodes) in which the immune response occurs.

By 13 weeks of gestation, each of the major T-cell subsets are present in cord blood and normally functional for mitogenic and allogeneic-stimulation.[6] However, fetal and neonatal T cells are virgin T cells because of the lack of prior exposure to antigen; this status explains the selective impairment of lymphokine production which likely contributes to the immune deficiency observed during the neonatal period: contrasting with a normal production of IL2, the production of other cytokines are either moderately (TNFa, GM-CSF) or markedly reduced (IL-3, IL-4, IL-5, IFN$\gamma$).[7–10] Moreover, neonatal T lymphocytes are less efficient for B cell help to immunoglobulin (Ig) synthesis, likely because of an impaired expression of the T-cell activation marker CD40-ligand.[11] During fetal life, infections can also occur when TCR repertoire is

*Correspondence to: A. Durandy, Institut National de la Santé et de la Recherche Médicale U429, Hôpital Necker-Enfants Malades, 149 rue de Sèvres 75743 Paris Cédex15

Received 1 October 1997

Accepted 21 October 1997

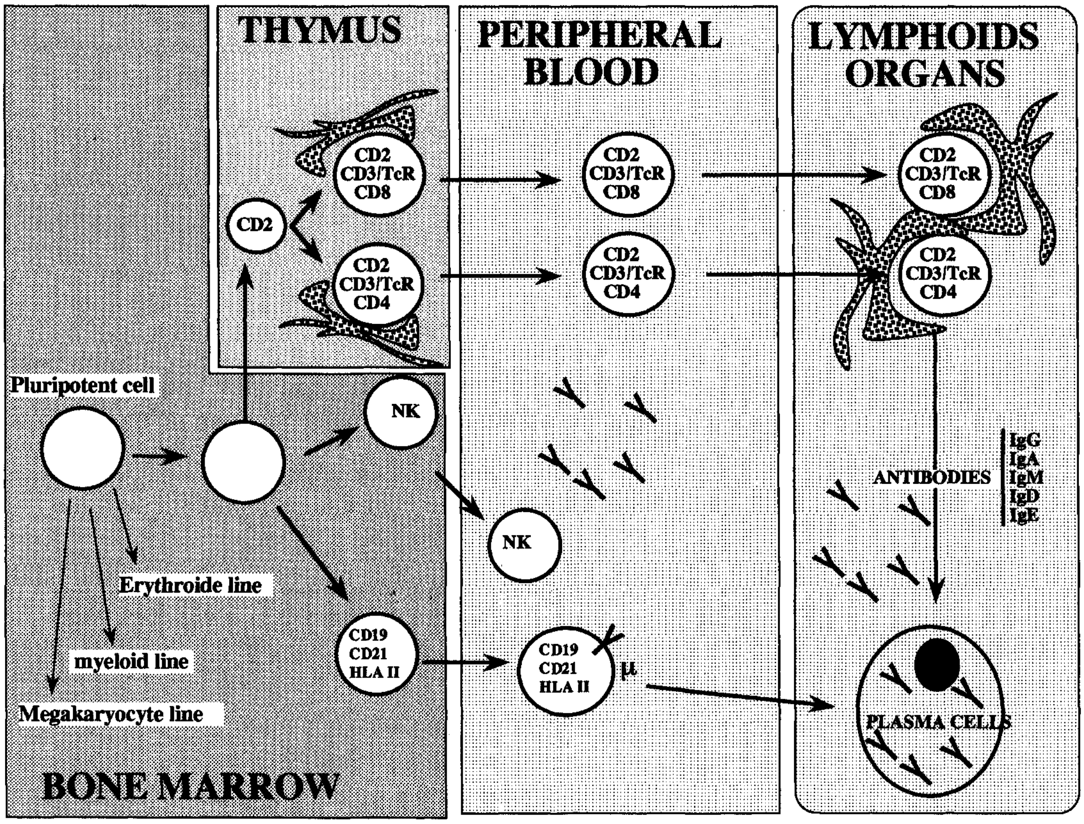

Fig. 1. Normal differentiation of T and B lymphocytes.

still limited; this situation can explain the severity of *in utero* early infections by *Toxoplasma gondii* or CMV.[2]

### Humoral Immunity

Precursors of B cells are first detected in fetal liver at 8 weeks of gestation and thereafter in bone-marrow.The maturation of pre-B cells into mature B cells occur in these hematopoietic organs. Immature B cells expressing a surface immunoglobulin (the B Cell Receptor or BCR) of the IgM isotype are detected at 12 weeks of gestation.[12] As for T cells, the diversity of the BCR repertoire is progressively acquired with positive and negative (deletion of auto-reactive B cells) selection mechanisms occuring in the bone-marrow. The main difference between TCR and BCR is the fact that BCR can recognize native antigens (and not processed antigenic peptides) without any interaction with HLA molecules. The mature B cell bearing on its membrane IgM+IgD leaves the bone-marrow and reaches the spleen and lymph nodes in which the immune response occurs. In germinal centers of lymphoid organs, other mechanisms take place, requiring a close relation with T cells: the immunoglobulin switching which allows a B cell to produce an immunoglobulin IgG, IgA or IgE with the same antigen specificity as the IgM previously produced and the positive selection of high affinity BCR bearing B cells.

Although specific antibody synthesis, essentially of the IgM isotype, is detectable as early as 20–24 weeks of gestation in an infected fetus, little IgM and IgA are present in the healthy neonate because of the lack of antigen exposure in normal conditions, and IgG is only from maternal origin (the transplacental transfer progressively increases from the 6th month of gestation). Immunoglobulin levels increase progressively to reach adult levels at 4 years of life. The neonate is able to produce specific antibodies to protein antigens but at a lesser degree than adults. This impaired response is likely related to the virgin status of B cells and to a defect in T-B cell cooperation. The response of infants to polysaccharidic antigens, a response which is supposed to be less T-cell dependent than the response to protein antigens, is severely impaired during the two first years of life.[13]

### NONSPECIFIC IMMUNITY DEVELOPMENT

### Natural Killer Activity

Cells with a Natural Killer (NK) phenotype are detected as early as 6 weeks of gestation in the fetal liver. NK activity increases progressively during fetal life; it is about 50% of adult NK activity at birth and reaches normal values at age of 4 years.[14]

### Antigen Presenting Cells

Macrophages are detectable very early (4 weeks of gestation) in the yolk sac and found in lymphoid organs at 13 weeks. However, at birth, their number in tissues appears reduced, especially in the lungs. This can be related to an impaired chemotaxis. The production of monokines has been reported as normal (IL-1) or slightly diminished (IL-6, TNFα and IL-8). The processing of native antigens and presentation to T cells are also efficient.[15]

### Polymorphonuclear Cells

The most obvious defect is the diminished storage pool and the impaired delivery of polymorphonuclear cells at sites of infections, a defect which can be reported to an impaired chemotaxis. Neutrophils precursors are first detected in the yolk sac at 6 weeks of gestation, then in the liver and bonemarrow. Functional mature polymorphonuclear cells are present at 12–14 weeks of gestation but represent a very low percentage of circulating white blood cells. Their number sharply increases after birth; however, preterm or severely infected neonates often present with a severe neutropenia.[16]

### Complement System

Synthesis of the different components of complement begins at the 8th week of gestation and progressively increases until birth. At that time, levels of the different factors are around 50% of adult values.[17]

### IMMUNE DEFICIENCIES

### Acquired Immunodeficiencies

Most of the abnormalities impairing the fetal immune system development are acquired *in utero*. It is especially the case for infections occuring during pregnancy, such as rubella, cytomegaolovirus (CMV) or human immunodeficiency virus (HIV). In these situations, T cells are defective both in their number and in their function.

Preterm neonates also present with a severe, but transitory, immune deficiency charaterized by a hypogammaglobulinemia, a neutropenia and a defect of the different complement factors.

An alloimmunisation of the mother directed against paternal antigens present on fetal polymorphonuclear cells may result in a severe, but transitory, neutropenia.

Finally, a defect of humoral immunity in the mother will result in a transitory hypogammaglobulinemia in the neonate.

### Inherited Immune Deficiencies

#### *Specific Immunity Deficiencies*

Numerous blockades can occur on the maturation pathway of T and/or B lymphocytes resulting in different inherited immunedeficiencies (Figure 2):

a = A defect of the lymphoid precursor results in a complete lack of T and B lymphocytes leading to a severe combined immunodeficiency (SCID), the transmission of which is autosomal recessive. An enzymatic defect (deficiency in the adenosine desaminase, an enzyme necessary for purine metabolism, the gene of which is located on chromosome 20) is found in around 50% of cases.[18]

b = In other cases, a defect of enzymes involved in TCR or Ig gene recombination is suspected.[19]

c = A selective defect of the T-cell precursors is defined by a complete absence of T lymphocytes with normal B cells. However, the B cells cannot produce antibodies without T-cell help. Thus, this immune deficiency is a SCID. One form is due to a mutation of the gene coding for a chain (the g chain) of several receptors for lymphokines involved in T-cell differentiation. This gene is located on chromosome X.[20] Another form, autosomal recessive, is secondary to a defect of an enzyme (*jak3*) which is known to transmit intracellular signals of the g chain.[21]

d = Other abnormalities can occur on the T-cell maturation pathway, including the defect of thymus. This immune deficiency, called Di George syndrome, is characterised by a complete absence of T cells (resulting in SCID) when the thymus is absent or by a partial T-cell defect in incomplete forms. The immune deficiency is associated with

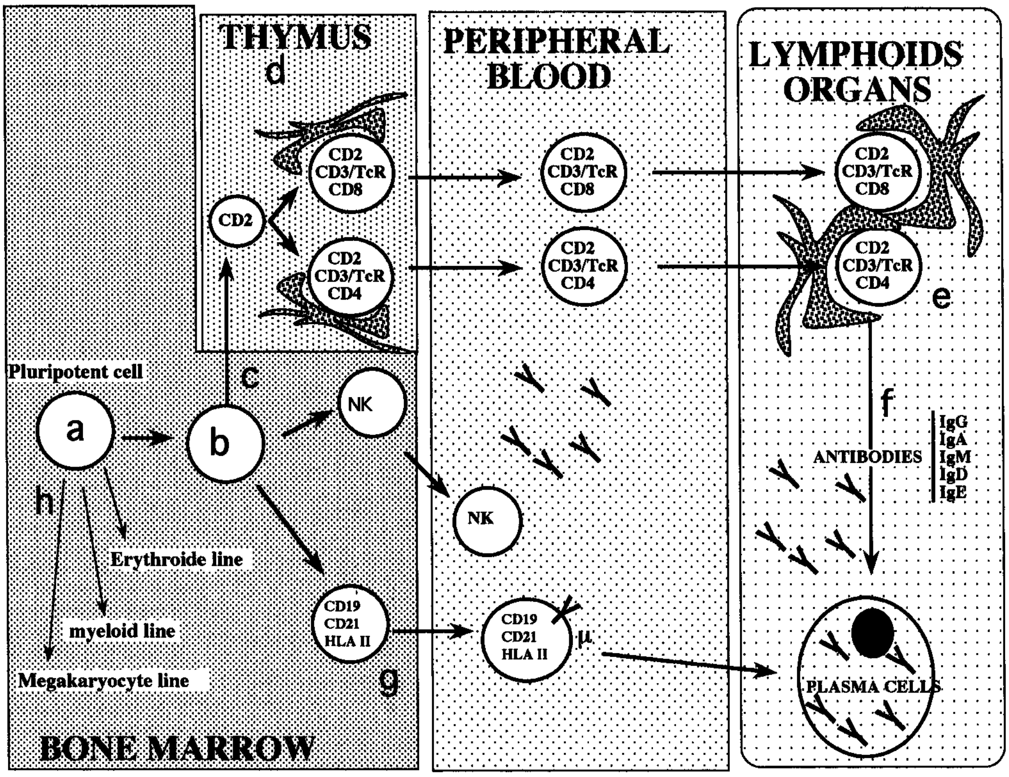

Fig. 2. Arrests of maturation of T and B lymphocytes.

cardiopathy and hypoparathyroidism. The mode of transmission is autosomal dominant, and a micro-deletion of chromosome 22 is constantly found.[22] e, f = Other abnormalities can also impair T-cell immunity development or function, as observed in defective expression of membrane antigens, resulting in combined immunodeficiencies: indeed, the defect in the T-cell pathway induces a secondary B cell deficiency. For example, in the X-linked hyper-IgM syndrome, a defect of expression of the molecule CD40-ligand on activated T cells (=f) results in a complete lack of immunoglobulin switching.[23] g = The B cell maturation pathway can also be affected, leading to a humoral immune defect. The best known is the X-linked agammaglobulinemia secondary to an early blockade on B lymphocyte differenciation. This abnormality has been reported to a defect in an enzyme (*btk*) necessary for B cell development.[24]

Two genes responsible when mutated for relatively frequent and severe immune deficiencies have been recently cloned: on X chromosome, the gene responsible for the Wiskott-Aldrich syndrome which associates an immune deficiency, eczema and thrombopenia (=h)[25] and on chromosome 11 the gene responsible for ataxia telangiectasia characterised by a T-cell defect and progressive neurological problems.[26]

### Nonspecific Immunity Deficiencies

Inherited defects can also impair nonspecific immunity: the chronic granulomatous disease is due to a genetic defect of cytochrome b involved in oxydative metabolism of polymorphonuclear cells. This disease is either X-linked or autosomal recessive, depending of the affected chain of cytochrome b.[27]

Other defects can affect the complement factors.

The recent cloning of different genes involved in maturation or function of specific or nonspecific immune cells has greatly improved our understanding of development of the immune system in normal human fetuses and neonates. In pathological conditions, this genetic approach allows a greater accuracy in postnatal diagnosis and is peculiarly useful for prenatal diagnosis. It also opens the way for gene therapy in the near future.

### REFERENCES

1. Durandy A. Developpement du système immunitaire. Pathol Biol 40:7–68. 1992.
2. English K, Wilson C: The Neonatal Immune Systeme in Clinical Immunology. Edition Mosby, 775, 1995.
3. Bach JF, Dardenne M, Papiernik A, Barois A, Levasseur PH, Le Brigand H: Evidence for serum factor secreted by the human thymus. Lancet II:1056–1062, 1972.
4. Lobach DF, Hensley LL, Ho W, et al.: Human T-cell antigen expression during the early stages of fetal thymic maturation. J Immunol 135:1752, 1985.
5. Haynes BF, Martin ME, Kay HH, Kurtzberg J: Early events in human T-cell ontogeny: phenotypic characterization and immunohistologic localization of T-cell precursors in early human fetal tissue. J Exp Med 168: 1061, 1988.
6. Durandy A, Dumez Y, Guy-Grand D, Oury C, Henrion R, Griscelli C: Prenatal diagnosis of severe combined immunodeficiency. J Pediatr 101:995–999, 1982.
7. Byrne JA, Butler JL, Cooper MD: Differential activation requirements for virgin and memory T cells. J Immunol 141:3249, 1988.
8. English BK, Burchett SK, English JD, et al.: Production of lymphotoxin and tumor necrosis factor by human neonatal mononuclear cells. Pediatr Res 24:717, 1988.
9. Wilson CB, Westall J, Johnston L, et al.: Decreased production of interferon-gamma by human neonatal cells. J Clin Invest 77:860, 1986.
10. English BK, Hammond WP, Lewis DB, et al.: Decreased granulocyte macrophage colony-stimulating factor (GM-CSF) production by human neonatal blood mononuclear cells and T cells. Pediatr Res 31:211, 1992.
11. Durandy A, De Saint Basile G, Gauchat J, et al.: Undectable CD40 ligand expression on T cells and low B cell responses to CD40 binding agonists in human newborns. J Immunol 154:1560–1568, 1995.
12. Lauwton AR, Self KS, Royal SA, Cooper MD: Ontogeny of B-lymphocytes in the human fetus. Clin Immunol Immunopathol 1:84–93, 1972.
13. Cowan MJ, Ammann AJ, Wara DW, et al.: Pneumococcal polysaccharide immunization in infants and children. Pediatrics 62:721, 1978.
14. Phillips JH, Hori T, Nagler A, et al.: Ontogeny of human natural killer ( NK) cells : fetal NK cells mediate cytolytic function and express cytoplasmic CD3ϵ, δ proteins. J Exp Med 175:1055, 1992.
15. Van Furth R, Raeburn JA, Van Zwet TL: Characteristics of human mononuclear phagocyte. Blood 54:485, 1979.
16. Hill HR: Biochemical, structural and functionnal abnormalities of polymorphonuclear leukocytes in the neonate. Pediatr Res 22:375, 1987.
17. Kohler PR: Maturation of the juman complement system 1. Onset time and sites of fetal C1q, C4, C3 and C5 synthesis. J Clin Invest 52:671, 1973.
18. Giblett ER, Anderson JE, Cohen F, et al.: Adenosine deaminase deficiency in two patients with severely impaired cellular immunity. Lancet 2:1067, 1972.
19. Schwarz K, Hausen-Hagge TE, Knobloch C, et al.: Severe combined immunodeficiency (SCID) in man. B cell-negative ($B^-$) SCID patients exhibit an irregular recombination pattern at the $J_h$ locus. .J Exp Med 174: 1039, 1991.
20. Noguchi M, Yi H, Rosenblatt HM, et al.: Interleukin-2 receptor $\gamma$ chain mutation results in X-linked severe combined immunodeficiency in humans. Cell 3:147, 1993.
21. Macchi P, Villa A, Giliani S, et al.: Mutations of Jak-3 gene in patients with autosomal severe combined immune deficiency (SCID). Nature 377:65–68, 1995.
22. Carey AH, Kelly D, Halford S, et al.: Molecular genetic study of the frequency of monosomy 22q11 in Di George syndrome. Am J Hum Genet 51:964, 1992.
23. Disanto JP, Bonnefoy JY, Gauchat JF, et al.: CD40 ligand mutations in X-linked immunodefiency with hyper IgM. Nature 631:541, 1993.
24. Vetrie D, Vorechowsky I, Sederas P, et al.: The gene involved in X-linked agammaglobulinemia is a member of the SRC family of protein tyrosine kinases. Nature 361:226–233, 1993.
25. Derry JM, Ochs HD, Franke U: Isolation of a novel gene mutated in Wiskott-Aldrich Syndrome. Cell 78: 635, 1994.
26. Kapp LN, Painter RB, Yu LC, et al.: Cloning of a candidate gene for ataxia telangiectasia group D. Am. J Hum Genet 51:45, 1992.
27. Ross D: The genetic basis of chronic granulomatous disease. Immunol Rev 138, 1994.
